# Increased Ventricular Premature Contraction Frequency During REM Sleep in Patients with Coronary Artery Disease and Obstructive Sleep Apnea

**Published:** 2008-11-01

**Authors:** Mari A Watanabe, Rajeshkumar Bhalodia, Eric J Lundequam, Peter P Domitrovich, Brian C Steinmeyer, Phyllis K Stein, Kenneth E Freedland, Stephen P Duntley, Robert M Carney

**Affiliations:** 1 Internal Medicine Department, St. Louis University (where the work was performed); 2 Heart Rate Variability Laboratory, Cardiovascular Division, Washington University; 3 Behavioral Medicine Center, Department of Psychiatry, Washington University; 4 Sleep Research Laboratory, Department of Neurology, Washington University

**Keywords:** sudden death, sleep, arrhythmia, autonomic nervous system, coronary disease

## Abstract

**Background:**

Patients with obstructive sleep apnea are reported to have a peak of sudden cardiac death at night, in contrast to patients without apnea whose peak is in the morning. We hypothesized that ventricular premature contraction (VPC) frequency would correlate with measures of apnea and sympathetic activity.

**Methods:**

Electrocardiograms from a sleep study of 125 patients with coronary artery disease were evaluated. Patients were categorized by apnea-hypopnea index (AHI) into Moderate (AHI <15) or Severe (AHI>15) apnea groups. Sleep stages studied were Wake, S1, S2, S34, and rapid eye movement (REM). Parameters of a potent autonomically-based risk predictor for sudden cardiac death called heart rate turbulence were calculated.

**Results:**

There were 74 Moderate and 51 Severe obstructive sleep apnea patients. VPC frequency was affected significantly by sleep stage (Wake, S2 and REM, F=5.8, p<.005) and by AHI (F=8.7, p<.005). In Severe apnea patients, VPC frequency was higher in REM than in Wake (p=.011). In contrast, patients with Moderate apnea had fewer VPCs and exhibited no sleep stage dependence (p=.19). Oxygen desaturation duration per apnea episode correlated positively with AHI (r^2^=.71, p<.0001), and was longer in REM than in non-REM (p<.0001). The heart rate turbulence parameter TS correlated negatively with oxygen desaturation duration in REM (r^2^=.06, p=.014).

**Conclusions:**

Higher VPC frequency coupled with higher sympathetic activity caused by longer apnea episodes in REM sleep may be one reason for increased nocturnal death in apneic patients.

## Background

Ventricular premature contractions (VPCs) are more common in patients with sleep-disordered breathing than in those without [1]. Although the risk associated with presence of VPCs is generally considered to be low [2], recent studies in subjects with no history of coronary artery disease have found that the risk of death and coronary events is 2-3 fold greater in subjects with VPCs compared to those without [3,4]. With regard to the specific risk for arrhythmic death, a study involving over 15,000 healthy men found that the presence of any VPC was associated with a 3- fold risk of sudden cardiac death [5]. Presence of complex VPCs  increases arrhythmic death risk further [2,5].

Gami et al reported in 2005 that patients found to have obstructive sleep apnea, had a peak in sudden cardiac death during the night, in striking contrast to the trough of sudden cardiac death during the night in patients without obstructive sleep apnea, and in the general population [6]. Heart rate turbulence is one of the most potent Holter electrocardiogram-based risk predictors for sudden cardiac death. It is calculated from heart rate fluctuations that follow a VPC [7]. Heart rate turbulence is a measure of baroreflex sensitivity and is reduced by increased sympathetic or decreased parasympathetic activity [8]. We recently found that heart rate turbulence values during the night were smaller than that expected from the parasympathetic dominance usually associated with sleep, and conjectured that VPCs arising during times of increased sympathetic activity, such as during rapid eye movement (REM) sleep, might be influencing the results [9]. The purpose of the current study was to identify differences between patients with and without obstructive sleep apnea in nocturnal VPC frequency, and the autonomic basis of these differences using heart rate turbulence measurements.

## Methods

Patients with known coronary artery disease were recruited for a study on depression and obstructive sleep apnea [10]. The study complied with the Declaration of Helsinki and was approved by the local ethics committee. Patients were excluded if they had a prior diagnosis of any sleep disorder. Additionally, patients were excluded if they were <19 weeks from coronary artery bypass graft or <6 months from acute myocardial infarction, were pacemaker dependent, or had left ventricular ejection fraction <35%.  Altogether, 1,167 patients were screened, 503 fulfilled enrollment criteria, and 134 patients consented. We obtained 125 analyzable records.

An obstructive apnea was defined as airflow cessation >10 seconds in presence of ventilatory effort. Hypopnea was defined as airflow reduction >30% and SaO2 reduction >3% for >10 seconds. The apnea-hypopnea index (AHI) was calculated as the sum of apnea and hypopnea events per hour. Patients were labeled as having No (AHI <5), Moderate (AHI 5 - 15), or Severe apnea (AHI>15). The term Severe was used to describe both AHI 15-30 and AHI>30 apnea, although some clinicians call the former moderate and the latter severe and AHI 5 - 15, mild apnea. Sleep stages were categorized as Wake, S1, S2, S34 (S3 or S4), and rapid eye movement (REM) according to standard EEG criteria [11], using 2-minute epochs in the same stage. The heart rate turbulence parameters turbulence slope (TS) and turbulence onset (TO), were calculated in the standard manner [9]. TS > 2.5 ms/beat and TO < 0% are considered normal.

All data are expressed as mean +/- SEM. A linear mixed model was fit to the data (SAS Institute, MIXED procedure) in order to obtain inference for the main effect of sleep stage (within-subjects factor), apnea severity (between-groups factor), and their interaction on each outcome. The effect of depression was assessed through the use of a continuous measure, the Beck Depression Inventory score.  A compound symmetry covariance structure was used to account for the intra-subject correlation that was inherent from sleep stage. A Tukey adjustment was applied to all post-hoc mean comparison tests. Correlation was assessed by univariate linear regression.  P < .05 was considered statistically significant.

## Results

### Patient and sleep stage characteristics

There were a total of 125 patients in the study; 37, 37, and 51 patients with No, Moderate, and Severe apnea, respectively. Twentyone of the 51 patients with Severe apnea had AHI >30. Patient characteristics (Table 1) were similar between patients with AHI < 15 or >15 with the exception of a greater number of men (p=.03) and greater number of patients on lipid lowering medications (p=.01) in the group with AHI>15. Depression score was similar for the two AHI groups,  consistent with previous results [10].

All patients had S2 and REM sleep stages of at least 2 minutes duration. There were 1, 12, and 32 patients in whom there were no valid recordings of Wake, S1, and S34 sleep stages, respectively. Two patients had neither valid S1 nor S34 recordings. Accordingly, there were 82 patients who had recordings in all sleep stages.

**Figure 1:**
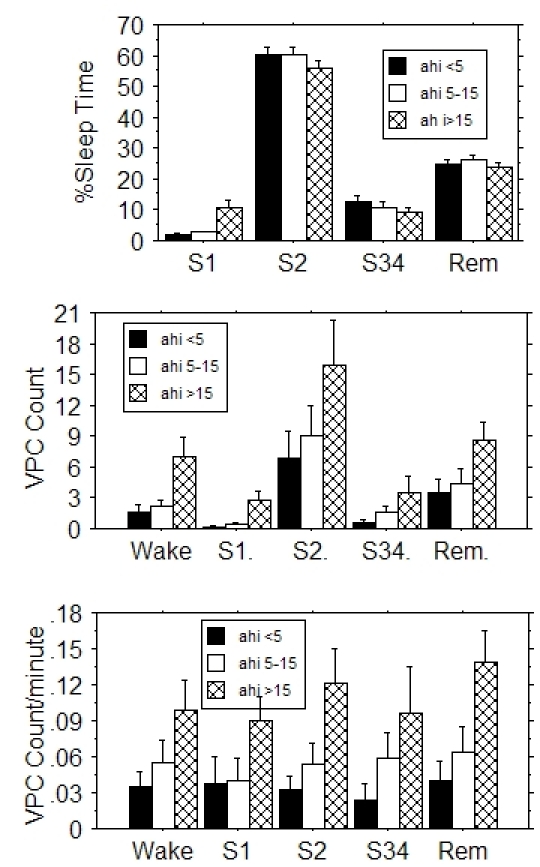
Sleep time as percent of total sleep time (top panel), VPC count per sleep stage (center panel) and VPC frequency (bottom panel) for the different sleep stages for patients with No, Moderate, or Severe obstructive sleep apnea as defined by apnea-hypopnea index (AHI) value. Error bars indicate SEM. Severe sleep apnea patients spent more time in S1 sleep than patients with No or Moderate apnea. Both VPC count and frequency increased with increasing severity of apnea.

Mean total sleep time was 302 ±12, 272 ±10, 255 ±10 minutes for patients with No, Moderate, and Severe sleep apnea, respectively. Total sleep time diminished as AHI increased (r^2^=.109, p<.001). When expressed as percent of total sleep time, approximately 60%, 25%, and 10% of time was spent in S2, REM, and S34 stages, respectively, regardless of apnea severity (Figure 1, top panel).

### VPC frequency

The number of patients with VPCs in a given sleep stage were Wake: 64, S1: 31, S2: 76, S34: 33, REM: 64. Twenty-five patients had no VPCs during any sleep stage: 9 in the No apnea, 7 in the Moderate, and 9 in the Severe apnea category. These patients did not differ from the 100 patients with at least one VPC with respect to the AHI (14 ±2.7 vs. 17 ±1.7, p=.33) or depression score [10] (17.9 ±2.3 vs. 14.0 ±1.1, p=.12). Only 12 patients had VPCs in all 5 sleep stages. There were 5 patients with > 30 VPCs per hour, and 33 patients with repetitive VPCs. The graph of total VPC count per sleep stage (Figure 1, middle panel) suggests that VPC count parallels time spent in a given sleep stage (Figure 1, top). We therefore computed VPC frequency, i.e., the number of VPCs per minute, including 0.0 as a VPC frequency if there were no VPCs (Figure 1, bottom). Regression analysis showed that VPC count and frequency both increased with increasing AHI (r^2^=.013, p<.001 and r^2^=.049, p<.0001, respectively).

**Figure 2:**
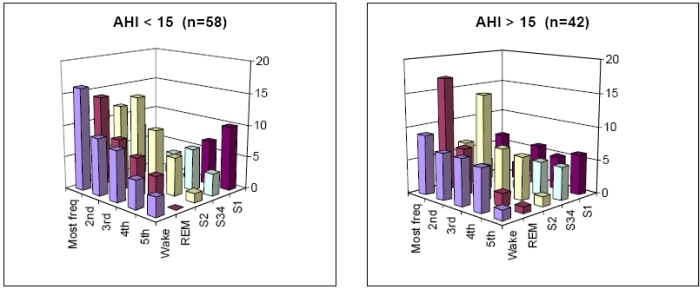
Histogram of sleep stage distribution of VPC frequency in patients with AHI <15 (left panel) and AHI>15 obstructive sleep apnea (right panel). In each patient, an ordered list of sleep stages was constructed from sleep stage with maximum VPC frequency to minimum VPC frequency. The ordering was tallied over all patients. The vertical axis shows number of patients. For example, the left panel shows Wake was the stage with the highest VPC frequency in 16 patients, whereas REM was the stage with the highest VPC frequency in 14 patients.

We next focused on possible differences in VPC frequency between sleep stages. To control for patients with high VPC frequency values who could be biasing VPC frequency results (bottom panel, Figure 1), we used VPC frequency to construct an ordered sleep stage list for each patient. For example, a patient whose highest VPC frequency was in REM, followed by S2, Wake, S1, and spent no time in S34, was represented by the ordered list: REM, S2, Wake, S1, blank. After creating this list for all patients, the number of each of the sleep stages in the first to last position in the list was tallied to construct a histogram (Figure 2). In patients with AHI<15 (n=58, left panel), the highest VPC frequency was found most often in Wake followed by REM, S2, S34 and S1, while conversely, the lowest VPC frequency was found most often in S1, followed by S34, Wake, S2, and REM. REM was never the sleep category with the lowest frequency of VPCs. The 3-dimensional graph shows a general reciprocal relationship: a smooth decrease in Wake and a smooth increase in S1 as VPC frequency decreases. The orderly appearance of this chart suggests VPC frequency dependence on sleep stage. Patients with AHI>15 (n=42, right panel) differed from patients with AHI<15 in that REM dominated the highest VPC frequency position.

We then studied results limited to the sleep stages of Wake, S2 and REM, because there were 124 patients who had records in all three stages. Sleep stage significantly affected VPC frequency (F=5.8, p=.0036). Post-hoc testing showed that VPC frequency was significantly greater for REM than for Wake (adjusted p=.0024), but similar for the other comparisons (p>0.13 for both). VPC frequency was greater for AHI>15 than for AHI<15 (F=8.7, p=.0039). There was borderline interaction between sleep stage and AHI dichotomized at 15 (p=.053). In post-hoc analysis based on the borderline p value, patients with AHI<15 had VPC frequency which was similar over the sleep stages (mean .047, n=74, F=1.7, p=.19), while in patients with AHI>15, VPC frequency differed across the sleep stages (mean .119, n=51, F=3.4, p=.037). Together with Figure 2, this suggested that the greater VPC frequency in REM than in Wake was a phenomenon of AHI>15 patients.

### VPC frequency and heart rate

The RR interval value preceding the VPC was used to assess heart rate. For AHI>15 patients, there was a correlation between VPC frequency and RR in S2 (r^2^=.31, p<.001) and REM (r^2^=.36, p<.001), and near correlation in S1 (r^2^=.16, p=.07). Patients with higher heart rate had higher VPC frequency except in Wake. Patients with AHI<15 showed no such correlations. There was no dependence of RR on AHI (r^2^=.008, p=.37) that could account for the difference between low and high AHI patients. RR limited to Wake, S2 and REM was also analyzed. Over all patients, there was a sleep stage dependence of RR (F=9.4, p=.0002). RR was greater for S2 (984 ±17 ms) than for REM (954 ±17 ms, adjusted p =.018) or for Wake (936 ±17 ms, adjusted p=.0001). REM and Wake RR did not differ, suggesting that the greater VPC frequency in REM over Wake found earlier was not due to heart rate.

### Heart rate turbulence

Of the 100 patients with VPCs, 5 had abnormal TS, and 17 had abnormal TO. Regression analysis showed there was no significant dependence of TS value (r^2^=.023, p=.13) or TO value (r^2^=.001, p=.71) on AHI. In the linear mixed model, AHI dichotomized at 15 and sleep stages limited to REM, S2 and Wake showed that TS was affected by sleep stage (F=5.4, p=.0058), but not by AHI (F=.03, p=.85). TS was higher in Wake and REM than in S2. TO was not affected by either of sleep stage (F=1.2, p=.30) or AHI (F=1.2, p=27). 

**Figure 3:**
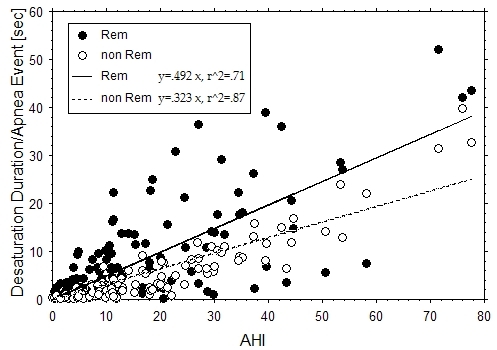
Correlation between oxygen desaturation duration/ apnea event and AHI. Duration of oxygen desaturation >2% (sec) from baseline per apnea event was more closely correlated with AHI and shorter in duration in non-REM sleep compared to REM sleep. Each patient is represented by one AHI value and two oxygen desaturation duration values, one for REM (filled circle) and one for non-REM (empty circle) sleep.

**Figure 4:**
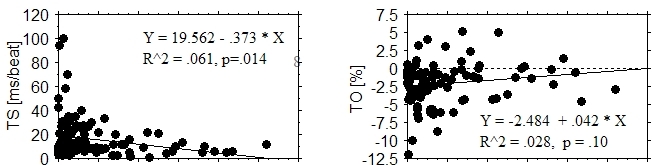
Relationship between heart rate turbulence values and oxygen desaturation duration per apnea event in REM stage sleep. Turbulence slope (TS) and turbulence onset (TO) were worse when the apnea duration was longer.

Although TS and TO values did not correlate with AHI (apnea *frequency*), they did correlate with measures of apnea *duration*. Oxygen desaturation duration (DesatDurn) per apnea event was plotted against AHI value for REM and non-REM (S1, S2, and S34) sleep (Figure 3). We observed that: (1) DesatDurn correlated significantly and positively with AHI (r^2^=.71, p <.0001 for REM; r^2^=.87, p<.0001 for non-REM). (2) On average, DesatDurn was longer in REM sleep (p<.0001). (3) DesatDurn was less well correlated with AHI in REM sleep. With respect to heart rate turbulence, TS values became more abnormal (decreased) (p=.014) as REM DesatDurn increased (Figure 4). There was no correlation between non-REM DesatDurn and either TS or TO (both p>.3). VPC frequency correlated with REM DesatDurn (p < .0001). Both TS and TO became more abnormal as relative DesatDurn increased (TS p<.01; TO p=.002), where relative DesatDurn was defined as REM DesatDurn - non-REM DesatDurn. Relative DesatDurn quantified the magnitude of DesatDurn in REM relative to that expected for a given AHI. Non-REM DesatDurn was convenient as a surrogate measure of AHI in having the same units as REM DesatDurn.

## Discussion

Novel findings: VPC frequency correlated positively with AHI. AHI>15 patients displayed higher VPC frequency in REM sleep than in Wake. Oxygen desaturation duration per apnea event was positively correlated with AHI, and was longer in REM than in non-REM sleep. The heart rate turbulence parameter TS correlated negatively with oxygen desaturation duration in REM sleep, and both turbulence parameters correlated with relative desaturation duration.

There have been previous studies of the relationship between sleep stage, VPC frequency, and sleep apnea duration. Our study is unique in comparing VPC frequency (Table 2, upper section) between Wake and sleep stages. With regard to apnea duration (lower section), the only previous study finding a significant relationship between apnea duration in REM and non-REM (S2) sleep was small and not limited to obstructive sleep apnea patients. The longer apnea duration in REM sleep may [16] or may not be due to greater airway collapsibility during REM sleep [17]. The correlation we found between apnea frequency and apnea duration implies that patients with more frequent apnea episodes also have longer episodes.

Heart rate immediately preceding a VPC was slowest in S2 followed by REM, then Wake, but did not differ statistically between REM and Wake. Neither was there an interaction between sleep stage and AHI with respect to heart rate. Therefore, heart rate dependence of VPCs did not cause the higher VPC frequency in REM compared to Wake. It is known that there are patients whose VPC frequency is heart rate dependent and decreases during sleep, and those whose VPC frequency is neither heart rate dependent nor affected by sleep [18,19].

A previous study of heart rate turbulence parameters in sleep apnea found a negative correlation between AHI and TS (r^2^=.20, p=.01) [20]. We found no correlation between AHI and TS. In contrast, the dependence of TS on oxygen desaturation duration we found is compatible with the pathophysiology of obstructive sleep apnea. Shepard et al found an inverse relationship between SaO2 and VPC frequency in the subset of patients whose average SaO2 during apnea was < 60% [12]. They also observed that VPCs occurred at the nadir of oxygen saturation or during its rapid recovery phase, when vagal withdrawal and sympathetic recruitment are prominent. Heart rate turbulence measured from VPCs at this phase should be smaller in magnitude, because heart rate turbulence is reduced by low vagal activity and high sympathetic activity [8]. Such observations lend support to the hypothesis that heart rate turbulence measured at night might be smaller in magnitude despite nighttime dominance of vagal activity, as a result of VPC occurrence at times of high sympathetic activity [9]. Patients with obstructive sleep apnea are known to have increased sympathetic activity, especially at night [21]. Thoracic efferents play a role in further increasing sympathetic activity during apnea [22].

### Clinical implications

Similar to others, we found that 41% of coronary artery disease patients with no suspected sleep disorders had AHI>15 [23,24]. This underscores the importance of considering the presence of this treatable disease in all coronary patients. A primary motivation for this study was to investigate why patients with obstructive sleep apnea might have a peak in sudden cardiac death at night [6]. We found higher VPC frequency in REM than in Wake in patients with AHI>15. We also found longer oxygen desaturation duration per apnea episode in REM sleep compared to non-REM sleep (all patients). Ostensibly benign VPCs likely serve as triggers of complex arrhythmias precisely when dominance of sympathetic over vagal activity during prolonged apnea episodes (as indicated by abnormal heart rate turbulence values) creates an autonomic milieu favoring perpetuation of the arrhythmia. Diagnosis and appropriate treatment of obstructive sleep apnea should therefore be beneficial in preventing sudden cardiac death. Treatment has been shown to decrease sympathetic activity [21] , ventricular ectopy [25], ventricular tachycardia [13], and in cases of severe obstructive sleep apnea, fatal and non-fatal cardiovascular events [26]. We note, however, that in patient populations referred for assessment of sleep apnea, in contrast to our patients with coronary artery disease but no suspected sleep disorders, apnea has not been found to increase ventricular arrhythmias [27,28].

### Limitations

We were unable to study the timing of VPCs relative to apnea events for technical reasons despite its obvious importance. Because the population of this study was selected for a depression study, the results presented here may not be representative of patients with coronary  disease. However, we found no correlations between apnea and depression. We did not measure ventricular wall stretch despite the importance of mechanical stimulation in arrhythmogenesis [29].

## Figures and Tables

**Table 1:**
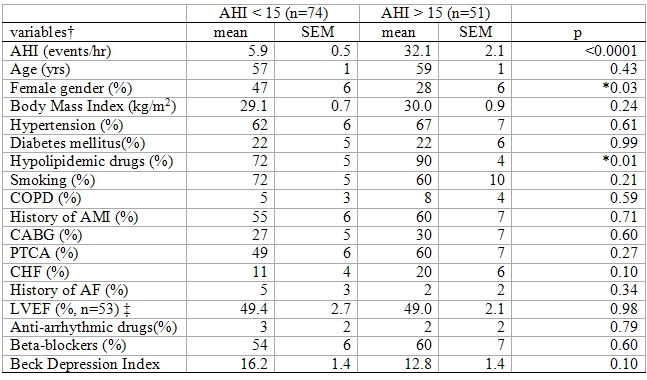
Patient characteristics by apnea severity

* significant difference between patient groups †AHI, age, Body Mass Index, LVEF and Beck Depression Index are measured values. All others are expressed as % of group positive for that feature. AF: atrial fibrillation, AMI: acute myocardial infarction, CABG: coronary artery bypass graft, CHF: congestive heart failure, COPD: chronic obstructive pulmonary disease, PTCA: percutaneous transluminal coronary angioplasty. ‡ LVEF data were available in 31 and 22 patients with AHI<15 or >15, respectively.

**Table 2:**
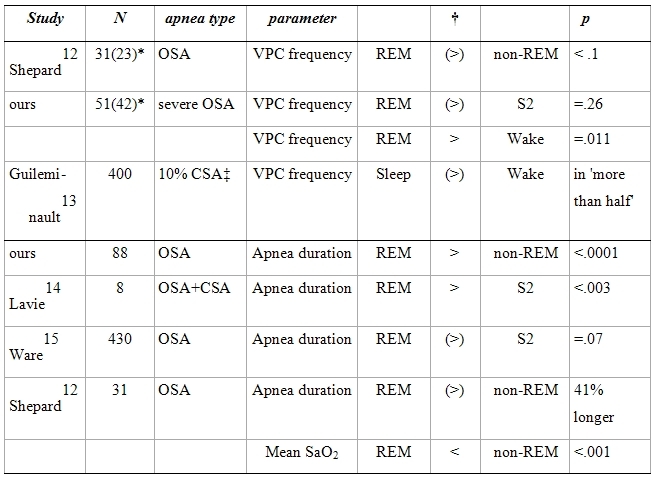
Studies of relationship between sleep stage and VPC frequency or apnea duration in patients with obstructive sleep apnea (OSA).

* Values in brackets indicate number of patients with at least one VPC. †Relational signs placed in brackets when p value not significant or not calculated. ‡ CSA: central sleep apnea.

## Glossary of abbreviations

VPC: ventricular premature contraction
AHI: apnea hypopnea index
TS: turbulence slope parameter of heart rate turbulence
TO: turbulence onset parameter of heart rate turbulence
RR: the time interval between two QRS peaks
